# Assessing the Effectiveness of Front of Pack Labels: Findings from an Online Randomised-Controlled Experiment in a Representative British Sample

**DOI:** 10.3390/nu13030900

**Published:** 2021-03-10

**Authors:** Jessica Packer, Simon J. Russell, Deborah Ridout, Steven Hope, Anne Conolly, Curtis Jessop, Oliver J. Robinson, Sandro T. Stoffel, Russell M. Viner, Helen Croker

**Affiliations:** 1 Population, Policy and Practice Research and Teaching Department, UCL Great Ormond Street Institute of Child Health, University College London, London WC1N 1EH, UK; s.russell@ucl.ac.uk (S.J.R.); d.ridout@ucl.ac.uk (D.R.); s.hope@ucl.ac.uk (S.H.); r.viner@ucl.ac.uk (R.M.V.); h.croker@ucl.ac.uk (H.C.); 2 National Centre for Social Research, London EC1V 0AX, UK; anne.conolly@natcen.ac.uk (A.C.); curtis.jessop@natcen.ac.uk (C.J.); 3 Institute of Cognitive Neuroscience, University College London, London WC1N 3AZ, UK; o.robinson@ucl.ac.uk; 4 Research Department of Behavioural Science and Health, University College London, London WC1E 6BT, UK; stoffsa@hotmail.com

**Keywords:** nutritional labelling, front of pack label, comprehension, randomised controlled experiment, nutrition policy

## Abstract

Front of pack food labels (FOPLs) provide accessible nutritional information to guide consumer choice. Using an online experiment with a large representative British sample, we aimed to examine whether FOPLs improve participants’ ability to identify the healthiness of foods and drinks. The primary aim was to compare ability to rank between FOPL groups and a no label control. Adults (≥18 years), recruited from the NatCen panel, were randomised to one of five experimental groups (Multiple Traffic Light, MTL; Nutri-Score, N-S; Warning Label, WL; Positive Choice tick, PC; no label control). Stratification variables were year of recruitment to panel, sex, age, government office region, and household income. Packaging images were created for three versions, varying in healthiness, of six food and drink products (pizza, drinks, cakes, crisps, yoghurts, breakfast cereals). Participants were asked to rank the three product images in order of healthiness. Ranking was completed on a single occasion and comprised a baseline measure (with no FOPL), and a follow-up measure including the FOPL as per each participant’s experimental group. The primary outcome was the ability to accurately rank product healthiness (all products ranked correctly vs. any incorrect). In 2020, 4504 participants had complete data and were included in the analysis. The probability of correct ranking at follow-up, and improving between baseline and follow-up, was significantly greater across all products for the N-S, MTL and WL groups, compared to control. This was seen for only some of the products for the PC group. The largest effects were seen for N-S, followed by MTL. These analyses were adjusted for stratification variables, ethnicity, education, household composition, food shopping responsibility, and current FOPL use. Exploratory analyses showed a tendency for participants with higher compared to lower education to rank products more accurately. Conclusions: All FOPLs were effective at improving participants’ ability to correctly rank products according to healthiness in this large representative British sample, with the largest effects seen for N-S, followed by MTL.

## 1. Introduction

**Figure 1:**
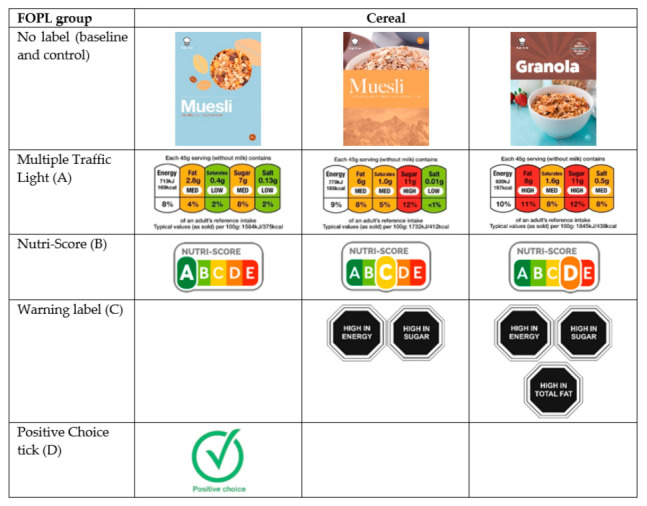
Example of the breakfast cereal products used for ranking tasks, with the associated front of pack labels.

Obesity continues to increase in many regions, and in young people, making it a key concern of policy makers [1]. Front of pack labels (FOPLs) are simplified representations of the detailed nutrient declarations found on the back of food packaging and are one of the recommended policy actions for improving diet and addressing obesity [2,3]. FOPLs aim to provide clearly visible and easy to use information, but they vary greatly in the type of information presented (numbers, percentages, text), design (colour, shape), creation (industry or Government), and implementation (mandatory or voluntary) [4]. They can be interpretive (providing information about how healthy a food is) or non-interpretive (where there is no judgement, such as reference intakes, RI). There are numerous examples of interpretive labels, including nutrient specific interpretive (e.g., UK Multiple Traffic Light, MTL, as per image A in Figure 1); summary indicators (e.g., French Nutri-Score, N-S, image B in Figure 1); nutrient-specific warnings (e.g., Chilean warning label, WL, image C in Figure 1); and endorsement logos (e.g., Healthy Choices, similar to the Positive Choice tick, PC, image D in Figure 1) [3].

FOPLs are proposed to improve dietary quality through two main pathways: improving consumer understanding (reliant on consumer engagement) and driving product reformulation [5]. Grunert and Wills present a theoretical framework describing factors involved in people’s responses to FOPLs, and propose that to be effective. A label must be seen, understood and liked by the consumer [6]. They also identify modifying factors that affect label effectiveness, including nutrition knowledge, interest in nutrition, demographics, and label format. Research shows that consumer acceptability of FOPLs is high and that FOPLs are more likely to be used and easier to understand than nutrition information on the back of pack [3,6,7]. Systematic reviews, including experimental and real-life settings, indicate that FOPLs improve the healthiness of product selection and purchases, and improve knowledge and ability to identify healthier products [8,9,10,11]. One review found that FOPLs can lead to health halo effects, with interpretive nutrient-specific labels found to improve health perceptions of both healthy and unhealthy products, although only influenced purchase intentions of healthy products [9]. There is limited direct evidence that FOPLs change intake [5,12,13]. Research indicates that interpretive labels have the most impact on consumer behaviour, especially simpler versions that provide an overall evaluation or use colour [3,9,11,13,14].

The influence of FOPLs on understanding is central to their effects and recent studies have examined this using ability to rank foods in order of “healthiness” as a marker of knowledge and understanding. An online experimental study in 12 countries tested the ability of participants to rank product healthiness, when randomised to one of five FOPL conditions (Health Star Rating, HSR; MTL; N-S; RI; WL) [15]. All FOPLs improved the ability of participants to correctly rank products, with the largest improvements seen for N-S, followed by the MTL, HSR, WL, and RI labels. An extension of this study with an additional six countries showed consistent results [16]. An online experimental study in Canada tested the ability of participants to rank food product healthiness, comparing MTL, HSR, WL and a no FOPL control [17]. All labels improved the ability of participants to identify and rank unhealthy products, with the HSR and MTL groups performing the best. Consumer engagement is essential for FOPLs to have an impact and research indicates that engagement is influenced by comprehension (nutritional knowledge) and motivation (such as interest in healthy eating), further highlighting the importance of understanding [7,18]. There is also evidence that socio-demographic (e.g., socio-economic status, age, sex, ethnicity) and contextual factors (e.g., food packaging, size, and other information included on the front of pack) impact engagement [3,8,9,19].

In 2014, the WHO recommended “interactive and consumer-friendly” FOPLs as a policy priority in the European Union (EU) [20]. A 2019 analysis of global FOPL regulation identified 32 governments endorsing FOPLs, 10 of which are mandatory [21]. The UK endorsed the MTL for voluntary use in 2013 [22]. Countries in the EU are constrained from implementing mandatory labels.

Now that the UK has left the EU, the UK Government has committed to review FOPLs to ensure that the country’s labelling scheme remains based on the latest evidence and to look at how MTL is implemented in the UK [23]. Although evidence to date consistently supports FOPLs as a tool to improve consumers’ ability to identify healthier foods and encourage healthier food purchasing, research in representative UK samples has been limited. This research sought to inform policy making around future FOPL options in the UK. We aimed to examine whether FOPLs were effective at improving participant understanding of the healthiness of foods and drinks in a large population-based British sample (including England, Scotland and Wales), and to explore whether this is influenced by level of education. Our primary objectives were to identify if FOPLs (MTL, N-S, WL, PC) performed better than no label controls, and to identify if FOPLs were effective at improving participants’ understanding of the healthiness of food items compared to no label. The main outcomes were the percentage of correct rankings (FOPLs vs. no label control), and the change in correct ranking from baseline to follow-up for food products individually and food products combined. Given the evidence supporting N-S, a secondary objective was to compare the effectiveness of N-S to the currently used MTL label at enabling an accurate product ranking. We also conducted exploratory analyses with education level and product ranking.

## 2. Materials and Methods

### 2.1. Study Design and Participants

This was an online experimental study, with participants randomised to one of four FOPL groups (MTL, N-S, WL, PC) or a no FOPL control, to examine whether FOPL type influenced ability to identify the healthiness of food products (used as a marker of understanding). The protocol and data analysis plan (the latter related to the primary and secondary objectives reported here) were specified a priori (see [24]). Data were collected 28th October- 15th November 2020. Participants were aged 18+, able to read and write in English and to complete the survey online. Participants were recruited from NatCen’s panel, a nationally representative, probability-based sample of adults in Great Britain (GB), i.e., England, Scotland and Wales [25]. Panel members (*n* = 7218) were contacted by letter, email and SMS with an invitation to participate and a link to the online survey and experiment. As an incentive, they were given a £5 Love2shop voucher on completion. The survey was created using NatCen’s established survey template, using UNICOM Intelligence software. Participants could complete the survey on computer devices, including smartphones, although were encouraged in the invitation email and prompted to switch to a device with a larger screen if phone used was detected by software.

Ethical approval was granted by NatCen’s Research Ethics Committee (application reference: P15640).

### 2.2. Materials

Six food and drink categories (pizza, instant hot chocolate, cake, crisps, yoghurt, breakfast cereal) were selected to cover a range of food types (ready-made meals, drinks, snacks, and breakfast cereals), each with adequate variability to create three nutritionally distinct products per category. The nutritional composition of the three products in each category were created to have a “most healthy”, middle and “least healthy” option, based on the 2004/5 UK Nutrient Profiling Model [26]. Mock images were created by a graphic designer to avoid brand loyalty and product familiarity effects of real products. They were standardised where possible (product weight, size) and had no nutritional claims, quality labels (e.g., organic) or allergen information. To create realistic products with hints of nutritional quality, design features such as packaging colour and product characteristics were used (e.g., “baked” to indicate “healthier” crisps, lighter colours to indicate “healthier” products), see Table S1 in the Supplementary File for full details. This approach was successfully used in previous experimental studies [15,16]. Established criteria were used to assign FOPLs based on each product’s nutritional profile. MTL and N-S label criteria were taken directly from technical guidance [27,28]. WL criteria were adapted from technical guidance, assigned to products with a “red” MTL label for that nutrient and energy WL if energy >275 kcal/100 g for food products and >70 kcal/100 mL for drinks (except no WL assigned to products that crossed energy thresholds but did not contain added sugars or saturated fat) [29]. The PC label was assigned where the food did not have any “red” MTL labels and did not exceed the energy threshold used for assigning the Chilean energy WL. The three products within each category were designed to have distinct label profiles, except for the PC label, which is binary. Public Health France (Santé Publique France) and Ministry of Health Chile (Ministerio de Salud) gave permission to use WL and N-S labels. The graphic designer created the MTL and PC labels. See Figure 1 for an example and Tables S1 and S2 in the Supplementary File for all product images, accompanying FOPLs and the full nutritional specification of the products.

### 2.3. Randomisation

NatCen used SPSS to randomly allocate equal numbers of participants to one of five experimental groups: MTL, N-S, WL, PC or control (no label). Randomisation was based on variables known before this study: year of recruitment to the panel; sex; age; government office region; and household income. Allocation was concealed from researchers and participants.

### 2.4. Procedure

We used a similar methodology and outcomes to a previous study [15]. Participants were told that they would be asked their views on food labelling on behalf of the UK Department of Health and Social Care. They were asked to complete 12 ranking tasks; each involved viewing three images of food products within a category and ranking them in order of healthiness, from most healthy to least healthy. The order of categories and of products within categories was randomised. We used a repeated-measures design; all participants completed the same 12 ranking tasks, six with no FOPLs (baseline) and six with packaging displaying the FOPL according to their assigned experimental group (follow-up). Participants could enlarge the images, but no other information (such as “back of pack” nutritional information) was provided. To minimise missing data, participants were only given a “don’t know” option if they tried to move onto the next page without completing the ranking task and were unable to rank two products the same.

### 2.5. Measures

#### 2.5.1. Primary Outcome

Our primary outcome was participants’ ability to accurately rank product healthiness. This was examined in three ways:

- ranking of healthiness of products (correct for all products = 1, any incorrect = 0) at baseline and follow-up;
- change in ranking from baseline to follow-up (improved = +1; no change = 0; worsened = −1);
- change in global food score, with change for each of the five food categories, i.e., excluding the drink (see Section 2.6), summed to give an overall score (range +5, all categories improved, to −5, all categories worsened).

Our primary and secondary objectives were based on these three outcomes.

#### 2.5.2. Other Secondary Outcomes

Participants were asked if they had enough information to complete each ranking task at follow-up (yes/no). They were also shown their assigned FOPL at follow-up and asked if they recalled seeing it (yes/no/not sure).

#### 2.5.3. Participant Characteristics

Several participant characteristics had been previously collected by NatCen (sex, age, ethnicity, educational level). Highest level of education was dichotomised for analysis into higher education (A-levels or vocational level 3 or equivalent and above, end of high school education in the UK) vs. lower education (below A-level or equivalent). We additionally collected data on household composition (children under 16 years), whether respondents were responsible for food shopping (yes/no), current FOPL use (very often/quite often/occasionally/rarely/never), and whether they had consumed or purchased each viewed food in the last 12 months (yes/no).

### 2.6. Statistical Analysis

Our sample size calculation was based on previous research using a similar methodology to the current study, we used the change in correct ranking of healthiness for the smallest relative change, where the percentage of participants who answered correctly for the pizza food group changed from 44% at baseline to 57% at follow-up [15]. Assuming a correlation between paired observations of 0.3, we calculated that a sample of 2400 (480 per group) would provide 90% power to detect differences of five percentage points, accounting for ineligibility, non-response, and design effects.

A multilevel log-binomial model was used to compare the proportion of correct rankings between baseline and follow-up. Similarly, we used a log-binomial model to compare the improvement rate in correct rankings from baseline to follow-up and a multiple linear regression analysis to compare the change in the global food score from baseline to follow-up. For these three outcomes, we compared each FOPL group with control, and made an additional comparison between MTL and N-S. All models were adjusted for the five stratification factors and the following pre-specified covariates: ethnicity, highest education level, household composition, food shopping responsibility, and current FOPL use. To ensure that lack of familiarity with products did not impact results, participants who reported they had not bought or consumed products from a particular product category in the last 12 months were excluded from analyses (see Table 1 for these numbers). For assessment of the change in global food score, only participants who reported buying or consuming all five food products were included. Given this requirement, the change in ranking of the drink was excluded from this analysis, as only 34% reported consuming/purchasing. All models were weighted to account for non-response and to ensure findings were representative of the British adult population. Analyses were not blinded, but were carried out by a statistician (DR) who had not been involved with setting up and running the experiment. Stata software (Release 16, StataCorp LLC., College Station, TX, USA) was used for all analyses and a significance level of 5% was used. Results are presented as relative risk (RR) of linear regression coefficient, with 95% confidence intervals.

We ran a sensitivity analysis fitting all models using the full sample of participants (*n* = 4530), irrespective of whether they consumed the particular food. Descriptive findings for the main outcome (correct/incorrect rankings at follow-up) are presented by level of education (higher vs. lower). Results at follow-up for reporting recalling seeing the FOPL and “having enough information to rank” are presented descriptively.

## 3. Results

**Figure 2:**
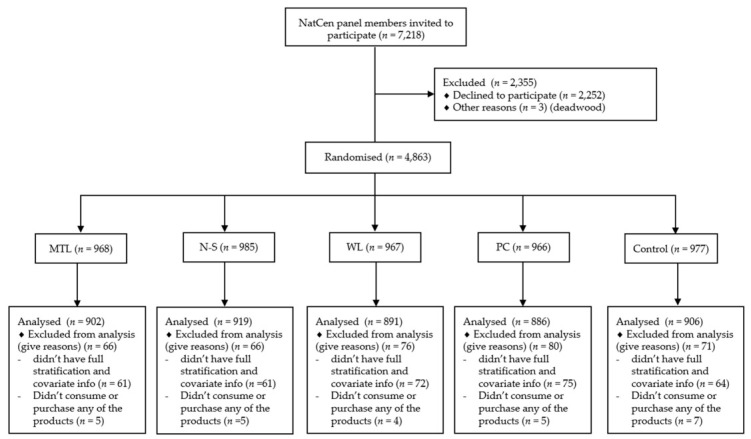
CONSORT flowchart. MTL: Multiple Traffic Lights; N-S: Nutri-Score; WL: Warning label; PC: Positive Choice tick.

Of the 7218 panel members contacted, 4863 agreed to participate (67% response rate) and were randomly allocated to: MTL (*n* = 968), N-S (*n* = 985), WL (*n* = 967), PC (*n* = 966) and control (*n* = 977). See CONSORT flowchart for more detail (Figure 2). The average time taken to complete the online survey was 13 min (SD 8 min), not including demographic data routinely collected by NatCen. Complete stratification and covariate information was available for 4530 participants, of which 4504 were included in this analysis. The number of missing data for the primary outcomes was very low (≤2% missing per variable, see Table S4 in the Supplementary File). The sample for each product category analysis differed slightly as inclusion was dependent on consuming/purchasing that specific product (see Tables S6–S8 in the Supplementary File for the sensitivity analyses using the full 4530 sample, results were consistent with the main analyses). The global food score sample was 1976, as inclusion was dependent on participants consuming/purchasing all five food products.

The individual participant characteristics are provided in Table 1 (see Table S3 in the Supplementary File for characteristics by experimental group). Baseline characteristics and sample sizes were similar across experimental groups. Overall, 57.1% of participants were female, 93.5% were White British or White Other, 48.5% had a degree or higher, 30.4% had children in the household, 95.8% had food shopping responsibilities, and 54.8% reported currently using food labels very or quite often. The proportion of participants consuming each product category was roughly equivalent across the five food groups.

The number of participants who recalled seeing the FOPLs and having enough information to rank the products is shown in Table 2. The response to seeing the FOPL was 74.4% overall; N-S had highest recall rate (87.4%), followed by WL (78.0%), MTL (76.9%) and PC (55.0%). Overall, across each of the products, the PC and control groups had the lowest proportion of participants reporting having enough information, followed by WL, N-S with MTL the highest.

**Figure 3:**
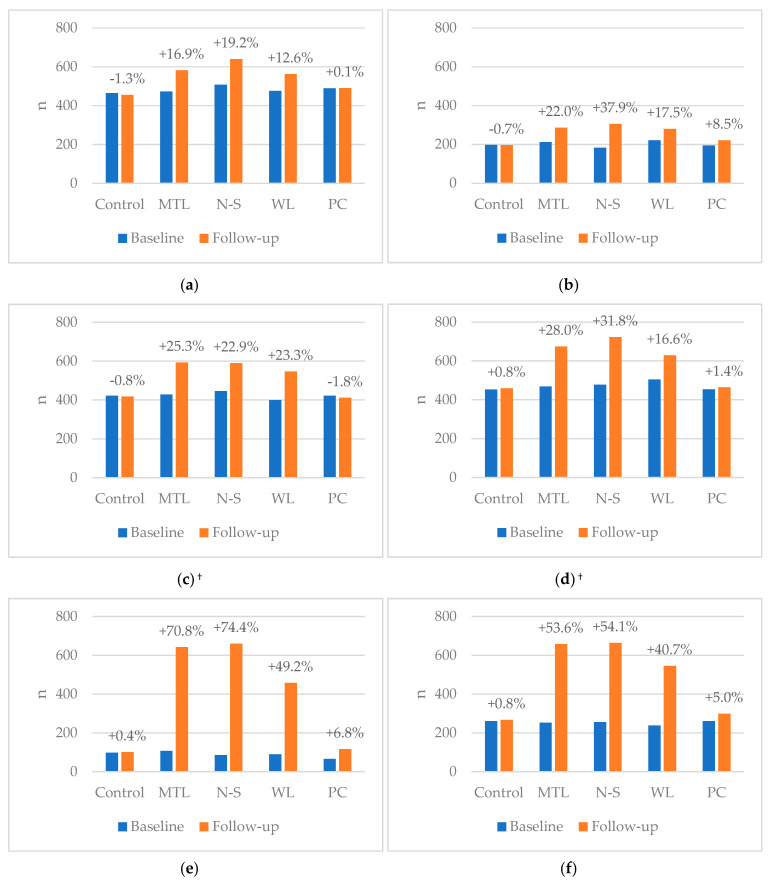
Number of participants who correctly ranked products at baseline and follow-up, by FOPL group and product category: (**a**) Pizza; (**b**) Drink; (**c**) Cake; (**d**) Crisps; (**e**) Yoghurt; (**f**) Breakfast cereal. ^†^ Cake and crisps categories had no products qualify for Positive Choice tick. MTL: Multiple Traffic Lights; N-S: Nutri-Score; WL: Warning label; PC: Positive Choice tick.

Figure 3 shows the percentage increase in correct ranking for each FOPL group, by food product. The proportions correctly ranking at baseline and follow-up are shown in Table S5 in the Supplementary File. The associations between FOPLs and correct product healthiness ranking at follow-up, adjusted for covariates, are shown in Table 3. We found that the probability of participants correctly ranking the products at follow-up was significantly greater across all products in N-S, MTL or WL groups, compared to control (all *p* < 0.001; except WL drink, *p* = 0.01). N-S was associated with the greatest probability of correct ranking followed by MTL, then WL and PC, compared to control. The PC group showed significant differences only for drink and yoghurt rankings. The comparison between N-S and MTL groups showed that participants in the N-S group were significantly more likely than the MTL group to correctly rank the drinks only.

The associations between FOPLs and improved change score, adjusted for covariates, are shown in Table 4. We found that N-S, MTL and WL significantly increased the probability of participants improving their score from baseline to follow-up, compared to control (all *p* < 0.001). The probability of improved ranking varied across N-S, MTL and WL groups, with the magnitude of RRs in that order. PC was associated with greater probability of improved score for only drinks, yoghurts, and breakfast cereals. The comparison between N-S and MTL showed that N-S significantly increased the probability of improving in score compared to MTL for drinks only.

The associations between global food score and FOPL groups, adjusted for covariates, are shown in Table 5. We found that N-S, MTL and WL were significantly associated with an increase in global food score compared to control (all *p* < 0.001), with the magnitude of effects in that order. No significant change was found for PC. The difference in global food score was small but significant between N-S and MTL.

Descriptive analyses of the proportion of participants correctly ranking products at follow-up, by experimental group and education level (higher vs. lower), are shown in Table 6. Across all experimental groups, and for all food and drink categories, a greater proportion of the participants with higher education correctly ranked the products at follow-up, compared to those with lower education. However, the difference between the two education groups varied considerably, with the N-S group appearing to have smallest differences.

## 4. Discussion

In this randomised controlled experiment, we found that, compared to a no FOPL control, all FOPLs significantly improved the ability of participants to correctly rank food and drink products according to healthiness. This was a consistent finding across all products and our three main outcomes, but the magnitude of the effects differed greatly between FOPL groups. The largest effects were seen for N-S, then MTL, WL and lastly, PC. The global food score analysis indicated that N-S led to improved rankings in an average of two product categories (2.1), compared to less than two categories for MTL (1.7) and WL (1.4) (see Table 5). We also found that there was limited learning between baseline and follow-up ranking in the control group, indicating that the experiment worked, the findings are reliable, and the observed effects were solely due to the inclusion of the FOPL.

These findings are consistent with previous studies, where N-S has out-performed other FOPLs, but literature is limited as the N-S is relatively new [15,16]. Results were similar in a Canadian study that included the HSR label (N-S and HSR are both summary indicator labels), suggesting that overall, these labels are more effective than the MTL label [17]. However, the differences between MTL and either N-S or HSR were greater than the current study. As evidenced in these studies and the current study, FOPLs consistently improve the ability to rank products [3,9,11]. All of the FOPLs in our study were interpretive, in that they provide consumers with some judgement of the healthiness of the food. These labels typically perform better than non-interpretive labels and those that are simple and use aids (such as colour) have been found to be the easiest and most likely to be used [3,9,13]. The use of colour could have contributed to the larger effects seen for N-S and MTL compared with WL and PC. N-S is a summary indicator label, which provides an overall nutritional assessment, whereas MTL provides nutrient-specific assessments, but no overall judgement. Others have found that interpretive labels providing an overall summary of healthiness are more helpful for consumers than those not providing a summary, but can lead to health halo effects [9,17].

The difference that we found between N-S and MTL appeared to be smaller than in other studies. This could be explained by participants being more familiar with the MTL, since the MTL has been in use in the UK since 2013 and understanding and knowledge have been shown to improve with familiarity with the label [3,30]. This could also impact on subjective understanding directly (whether consumers think they have “understood” the information from the label) and through subconscious perception (exposure to familiar concepts leads to subconscious activation) [6]. Such factors could also have contributed to the larger effects seen for the MTL label than the WL and PC labels. Concerns over the accuracy of FOPL have been found to have a negative impact on use, and this could have reduced effects for less well-known labels such as N-S, WL, and PC. Overall, 75% of participants recalled seeing the FOPL that they were randomised to, N-S had the highest recall and PC the lowest; this is significant, as the perception of labels is a key factor in engagement, as highlighted in the Grunert and Wills conceptual framework [6]. PC was least effective across the three outcomes; this is unsurprising, since endorsement labels are binary so provide limited information and two of the products did not qualify for any PC. Participants in MTL group scored highest for reporting “having enough information to rank”, despite N-S group generally performing better. This finding highlights the difference between objective understanding (outcomes of the ranking task) and subjective understanding (perceiving that they understood), where N-S resulted in highest objective but not highest subjective understanding.

Differences between product categories showed that yoghurt and breakfast cereal categories had the greatest improvements in correct ranking from baseline to follow-up, compared to the other foods. This shows that FOPL effectiveness might differ depending on product type and context or motivation for buying, such as hedonic pleasure [31]. Both of these products may also be perceived as “healthy” products and often feature health claims (e.g., “light”, “low-fat”). Health claims (which were not included in the mock images) can bias opinions of food, which FOPLs have shown potential in reducing [10]. These factors may explain the difference in responses and highlight the potential effectiveness of mandatory labels in these seemingly confusing products.

FOPLs’ effects were independent of SES, ethnicity, age, sex, household composition, food shopping responsibility, and current FOPL use. There was, however, a tendency for participants with higher education to be able to rank products with greater accuracy than those with less education. N-S seemed to show the smallest difference between groups with higher and lower education levels compared to other FOPL groups (especially marked for pizza, drinks, cake, and crisps, less so for yoghurts and breakfast cereals). In contrast to previous reports, the results from our study suggest some differences in responses to FOPL according to the level of education, with N-S appearing better in those with lower education levels. However, this was not the case for all foods, and as we did not statistically test differences, it warrants further investigation.

The findings from this study support FOPLs as a policy intervention to improve British consumers’ ability to understand the healthiness of food products. Our findings provide evidence that both the MTL and N-S FOPLs are likely to be most effective at improving knowledge. Whilst there was some evidence that N-S had the largest effects, the difference between MTL and N-S was relatively small and not significant for individual foods.

The MTL system was introduced in 2013 and the UK government is currently considering whether it remains the best FOPL system [30]. Our study provides evidence to inform this review, but any changes will also depend on how strongly the MTL is embedded in UK food policy. It is important that policies to improve food choices do not disproportionately affect individuals from more deprived or less educated backgrounds. Our findings suggest that there are benefits for all individuals regardless of education level, but there were some indications that N-S was most effective for those with a lower education. Caution is needed when interpreting these findings, since these were descriptive analyses. Knowledge can support people in making healthier choices, and evidence from reviews has shown that FOPL has the potential to encourage healthier purchases [13]. To make use of FOPLs, shoppers first need to notice and engage with the system. This is easier if they like the label and its format [6]. Participants in the current study had high recall for all the labels, except the PC. Other factors, such as context (for example, time, shopping location, price, marketing) and previous purchase and product preference also influence purchase decisions [31]. Consistent use of FOPLs on packaging (as would be the case with a mandatory system) and trust in their accuracy are also key factors [32]. Public education campaigns would help.

Our study was high quality, appropriately powered, with pre-specified protocol and analysis plans, which extended previous findings. It involved a large representative sample of adults in GB, included a control group and was broadly comparable to the full NatCen panel and the British population [25,33]. There were, however, some limitations to the design. The PC label does not provide sufficient information to rank three products, as it is a binary label, and two food categories had no products that qualified for it, as would be expected for this particular label. This is indicative of real-world issues related to PC. Our analysis sample for global food score was limited to 43.6% (*n* = 1976), as we pre-specified that we would only include participants who had consumed or purchased the product within the last 12 months. Due to the low level of instant hot chocolate consumption or purchase, this category was excluded from global score. Sensitivity analyses showed that results were stable when we considered all participants. We created mock images of all products to reduce bias and variability, but this may have reduced ecological validity. We did not include the back of pack information, as some studies have done, as we wanted the ranking to be exclusively informed by FOPL. In a real-life context, people would have access to additional information, so our limited information may have underestimated their ability to rank products. However, this is unlikely to be substantial, as evidence shows that people rarely read the back of pack [3,6]. The proportion of participants correctly ranking the products at baseline varied greatly across food products and was markedly low for yoghurt. This led to large increases in correct ranking at follow-up for this category and indicates that yoghurts were harder to rank without an FOPL. Despite all images being designed to allow correct ranking, we cannot ignore the possibility that the yoghurt images may have been inappropriate and difficult for participants to rank.

## 5. Conclusions

This study shows that FOPLs can be effective to improve knowledge of product healthiness in a large representative British sample. The N-S label had the largest effect on knowledge, closely followed by MTL and then WL labels. PC labels had the smallest effect, but all labels were more effective than the no label control. This study provides important evidence on FOPL, which is currently limited in the UK context, and this can inform future policy decisions.

## Figures and Tables

**Table 1:** Individual characteristics of the analysis sample (*n* = 4504; unweighted).

	*n* (%)
Sex	
Female	2570 (57.1)
Male	1934 (42.9)
Age *	
18–29	278 (6.2)
30–39	639 (14.2)
40–49	878 (19.5)
50–59	962 (21.4)
60–69	924 (20.5)
70+	820 (18.2)
Ethnicity	
White British	3954 (87.8)
White other	257 (5.7)
Mixed or multiple ethnic groups	54 (1.2)
Asian or Asian British	152 (3.4)
Black or Black British	71 (1.6)
Other	16 (0.4)
Education	
Degree or equivalent	2182 (48.5)
A-levels or vocational level 3 or equivalent	874 (19.4)
Other qualifications below A-levels or equivalent	781 (17.3)
Other qualification	269 (6.0)
No qualifications	398 (8.8)
Children in household	
Yes	1373 (30.4)
No	3131 (69.6)
Shopping responsibility	
Yes—some or all	4316 (95.8)
No—someone else does	188 (4.2)
Current label use	
Very often	934 (20.7)
Quite often	1536 (34.1)
Occasionally	1316 (29.2)
Rarely	558 (12.4)
Never	160 (3.6)
Reported consuming or buying product in past 12 months	
Pizza	3361 (74.6)
Drink	1630 (36.2)
Cake	3163 (70.2)
Crisps	3723 (82.7)
Yoghurt	3779 (83.9)
Breakfast cereal	3802 (84.4)

* adds up to 4501.

**Table 2:** Results of post-ranking questions at follow-up across the study sample, (*n* = 4504).

Reported Seeing FOPLs * (*n* = 3598)						
	Overall	MTL	N-S	WL	PC	Control *
Yes	2679 (74.4)	693 (76.8)	803 (87.4)	695 (78.0)	488 (55.1)	*n*/a
No/not sure	919 (25.6)	209 (23.2)	116 (12.6)	196 (22.0)	398 (44.9)	*n*/a
**Reported having enough information to rank (*n* = 4504) (Yes response)**						
	Overall	MTL	N-S	WL	PC	Control
Pizza	1351 (40.2)	550 (85.0)	405 (59.0) ^†^	243 (35.2) ^†^	67 (10.0)	86 (12.8)
Drink	611 (37.5)	276 (82.1)	180 (55.6)	111 (33.0)	22 (6.9) ^	22 (7.0) ^†^
Cake	1362 (43.1)	546 (83.9) ^†^	379 (59.9)	279 (43.9)	70 (11.4)	88 (14.0)
Crisps	1651 (44.4)	647 (87.6)	472 (61.1)	317 (42.1)	93 (13.2)	122 (16.2)
Yoghurt	1494 (39.5)	647 (85.7)	444 (57.5)	231 (30.9) ^†^	86 (11.6) ^†^	86 (11.3)
Breakfast cereal	1533 (40.3)	649 (85.7)	455 (60.3) ^	253 (33.5) ^†^	87 (11.5)	89 (11.4) ^†^

* Control group not applicable; ^†^ Does not equal 100% due to: 1 Don’t know response (*n* = 1, 0.1%); ^ or 2 Don’t know responses (*n* = 2, 0.6%). MTL: Multiple Traffic Lights; N-S: Nutri-Score; WL: Warning label; PC: Positive Choice tick.

**Table 3:** Multilevel log-binomial regression results—follow-up (FOPL group) correct (yes/no) and adjusted for baseline rank compared to control (also adjusted for design effects and covariates as planned).

	MTL vs. Control RR (95%CI)	N-S vs. Control RR (95%CI)	WL vs. Control RR (95%CI)	PC vs. Control RR (95%CI)	N-S vs. MTL RR (95%CI)
Pizza	1.23 (1.11, 1.36) *p* < 0.001	1.29 (1.18, 1.41) *p* < 0.001	1.20 (1.09, 1.32) *p* < 0.001	0.98 (0.90, 1.07) 0.69	1.05 (0.95, 1.16) *p* = 0.32
Drink	1.29 (1.13, 1.46) *p* < 0.001	1.61 (1.41, 1.84) *p* < 0.001	1.16 (1.03, 1.30) *p* = 0.01	1.18 (1.04, 1.33) *p* < 0.01	1.25 (1.08, 1.46) *p* < 0.01
Cake	1.47 (1.32, 1.63) *p* < 0.001	1.42 (1.29, 1.57) *p* < 0.001	1.38 (1.25, 1.52) *p* < 0.001	1.04 ^†^ (0.96, 1.12) 0.38	0.97 (0.87, 1.08) *p* = 0.55
Crisps	1.45 (1.32, 1.59) *p* < 0.001	1.48 (1.35, 1.62) *p* < 0.001	1.26 (1.16, 1.37) *p* < 0.001	1.03 ^†^ (0.95, 1.11) 0.50	1.02 (0.92, 1.14) *p* = 0.70
Yoghurt	5.72 (4.30, 7.59) *p* < 0.001	6.86 (4.90, 9.60) *p* < 0.001	4.22 (3.02, 5.90) *p* < 0.001	1.73 (1.25, 2.38) *p* < 0.01	1.20 (0.83, 1.74) *p* = 0.34
Breakfast cereal	2.48 (2.09, 2.96) *p* < 0.001	2.61 (2.18, 3.12) *p* < 0.001	2.35 (1.93, 2.88) *p* < 0.001	1.14 (0.95, 1.36) *p* = 0.15	1.05 (0.87, 1.27) *p* = 0.60

^†^ Cake and crisps categories had no products qualify for Positive Choice tick; all analyses were adjusted for baseline ranking (correct/incorrect), stratification factors (year of recruitment to panel, sex, age, government office region, household income), and the following pre-specified covariates: ethnicity, highest education level, household composition, food shopping responsibility, and current FOPL use. MTL: Multiple Traffic Lights; N-S: Nutri-Score; WL: Warning label; PC: Positive Choice tick; RR: Relative Risk; CI: Confidence Interval.

**Table 4:** Log-binomial regression results–relative risk that ranking improved (follow-up vs. baseline) between FOPL group and control (adjusted for design factors and covariates as planned).

	MTL vs. Control RR (95%CI)	N-S vs. Control RR (95%CI)	WL vs. Control RR (95%CI)	PC vs. Control RR (95%CI)	N-S vs. MTL RR (95%CI)
Pizza	2.66 (1.72, 4.11) *p* < 0.001	2.70 (1.76, 4.15) *p* < 0.001	2.36 (1.51, 3.67) *p* < 0.001	0.90 (0.56, 1.46) 0.67	1.02 (0.78, 1.33) *p* = 0.90
Drink	4.27 (2.36, 7.74) *p* < 0.001	6.10 (3.43, 10.84) *p* < 0.001	2.95 (1.61, 5.41) *p* < 0.001	2.37 (1.17, 4.77) *p* < 0.001	1.43 (1.04, 1.96) *p* = 0.03
Cake	6.89 (3.80, 12.50) *p* < 0.001	6.21 (3.46, 11.14) *p* < 0.001	5.60 (3.11, 10.10) *p* < 0.001	0.91 ^†^ (0.41, 2.02) 0.82	0.90 (0.70, 1.15) *p* = 0.40
Crisps	5.28 (3.59, 7.76) *p* < 0.001	5.83 (3.97, 8.56) *p* < 0.001	3.69 (2.46, 5.53) *p* < 0.001	1.40 ^†^ (0.82, 2.40) 0.22	1.10 (0.90, 1.35) *p* = 0.34
Yoghurt	22.36 (13.77, 36.31) *p* < 0.001	22.80 (14.04, 37.04) *p* < 0.001	15.81 (9.69, 25.81) *p* < 0.001	2.92 (1.65, 5.16) *p* < 0.001	1.02 (0.93, 1.12) *p* = 0.68
Breakfast cereal	7.03 (4.96, 9.98) *p* < 0.001	7.75 (5.47, 10.97) *p* < 0.001	6.43 (4.51, 9.17) *p* < 0.001	1.95 (1.29, 2.95) *p* < 0.01	1.10 (0.97, 1.25) *p* = 0.13

^†^ Cake and crisps categories had no products qualify for Positive Choice tick; all analyses were adjusted for the five stratification factors (year of recruitment to panel, sex, age, government office region, household income) and the following pre-specified covariates: ethnicity, highest education level, household composition, food shopping. MTL: Multiple Traffic Lights; N-S: Nutri-Score; WL: Warning label; PC: Positive Choice tick; RR: Relative Risk; CI: Confidence Interval.

**Table 5:** Multiple regression analysis results—association between global food score and FOPL group (adjusted for design factors and covariates as planned).

	MTL vs. Control RR (95%CI)	N-S vs. Control RR (95%CI)	WL vs. Control RR (95%CI)	PC vs. Control RR (95%CI)	N-S vs. MTL RR (95%CI)
Score (−5, +5) Regression (coefficients)	1.7 (1.6, 1.9) *p* < 0.001	2.1 (1.9, 2.2) *p* < 0.001	1.4 (1.3, 1.6) *p* < 0.001	0.1 (−0.02, 0.3) 0.09	0.3 (0.2, 0.5) *p* < 0.001

Global food score was an aggregated score of correct ranking in the five food products, range −5 to +5 (− indicates worsening and + indicates improvement); all analyses adjusted for stratification factors (year of recruitment to panel, sex, age, government office region, household income) and covariates: ethnicity, highest education level, household composition, food shopping responsibility, current FOPL use. MTL: Multiple Traffic Lights; N-S: Nutri-Score; WL: Warning label; PC: Positive Choice tick; RR: Relative Risk; CI: Confidence Interval.

**Table 6:** Proportion of participants correct (yes/no) at follow-up, by FOPL group and education level (higher/lower).

Education	FOPL Group				
	MTL	N-S	WL	PC	Control
**Pizza**					
Higher	427/460 (92.8%)	462/493 (93.7%)	411/479 (85.8%)	358/478 (74.9%)	333/474 (70.3%)
Lower	155/187 (82.9%)	178/193 (92.2%)	152/211 (72.0%)	133/190 (70.0%)	123/196 (62.8%)
**Drink**					
Higher	209/240 (87.1%)	232/245 (94.7%)	205/242 (84.7%)	160/230 (69.6%)	143/226 (63.3%)
Lower	77/96 (80.2%)	74/79 (93.7%)	75/94 (79.8%)	61/91 (67.0%)	53/87 (60.9%)
**Cake**					
Higher	406/439 (92.5%)	426/451 (94.5%)	383/437 (87.6%)	302/434 (69.6%)	303/429 (70.6%)
Lower	187/212 (88.2%)	164/182 (90.1%)	164/198 (82.8%)	109/183 (59.6%)	114/198 (57.6%)
**Crisps**					
Higher	468/500 (93.6%)	503/533 (94.4%)	442/520 (85.0%)	312/477 (65.4%)	325/526 (61.8%)
Lower	207/239 (86.6%)	220/239 (92.1%)	187/233 (80.3%)	152/230 (66.1%)	134/226 (59.3%)
**Yoghurt**					
Higher	452/521 (86.8%)	481/545 (88.3%)	327/515 (63.5%)	86/520 (16.5%)	75/532 (14.1%)
Lower	190/234 (81.2%)	179/227 (78.9%)	131/232 (56.5%)	31/224 (13.8%)	26/229 (11.4%)
**Breakfast cereal**					
Higher	460/503 (91.5%)	477/512 (93.2%)	380/510 (74.5%)	224/512 (43.8%)	191/532 (35.9%)
Lower	198/254 (78.0%)	187/243 (77.0%)	166/246 (67.5%)	75/243 (30.9%)	76/247 (30.8%)

Education level dichotomised as A-levels and above vs. below A-levels, higher or lower education respectively. MTL: Multiple Traffic Lights; N-S: Nutri-Score; WL: Warning label; PC: Positive Choice tick.

## Data Availability

The data presented in this article are available on reasonable request from the corresponding author.

## Supplementary Materials

The following are available online at https://www.mdpi.com/2072-6643/13/3/900/s1, Table S1: Product images, applicable labels and design features, Table S2: Nutritional specification of products. Table S3: Individual characteristics of the analysis sample, by experimental group, Table S4: Proportion of missing data per variable, Table S5: Summary of participants who correctly ranked products at baseline and follow-up, by FOPL group and product category, Table S6: Multilevel log-binomial regression results-follow-up (FOPL group) correct (yes/no) and adjusted for baseline rank compared to control in full sample (*n* = 4530), Table S7: Log-binomial regression results–relative risk that ranking improved (follow-up vs. baseline) between FOPL group and control full sample (*n* = 4530), Table S8: Multiple regression analysis results- association between global food score and FOPL group in full sample (*n* = 4530).
