# Supplementary material for: Assessing the Effectiveness of Front of Pack Labels: Findings from an Online Randomised-Controlled Experiment in a Representative British Sample
Source: Nutrients. 2021 Mar 10;13(3):900. doi: 10.3390/nu13030900 (PMC7999818; doi:10.3390/nu13030900)
Supplement: Supplementary file 1 [file nutrients-13-00900-s001.pdf]

Supplementary File

Table S1. Product images, applicable labels and design features

**FOPL group**	**Product**		
	**Pizza**		
**No label (baseline and control)**	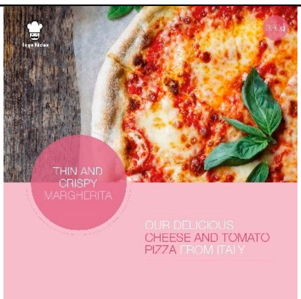	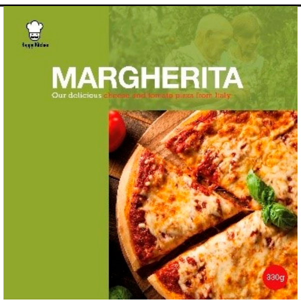	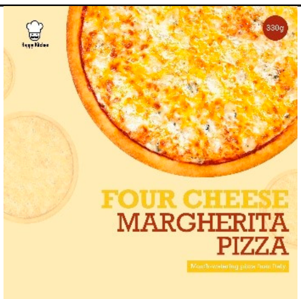
**Multiple Traffic Light**	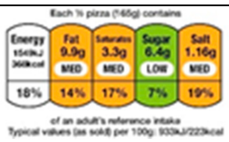	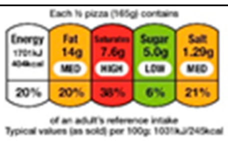	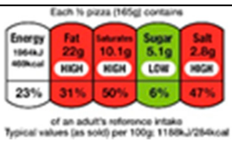
**Nutri-Score**	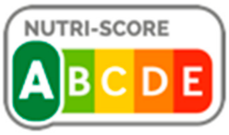	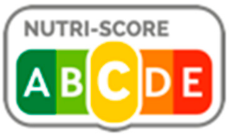	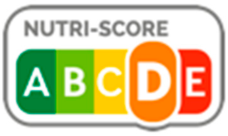
**Warning label**		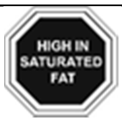	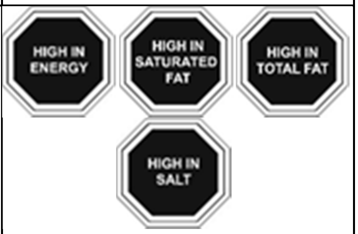
**Positive Choice tick**	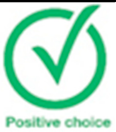		
**Design features**<br><br>All: size=330g (standard pizza box)	Description: 'Thin and crispy margherita' and 'Our delicious cheese and tomato pizza from Italy'<br>Image: Pizza with cheese, tomato sauce and fresh basil<br>Colour: light pink	Description: 'Margherita' and 'Our delicious cheese and tomato pizza from Italy'<br>Image: Pizza with cheese (more than 1), tomato sauce and fresh basil (less than 1)<br>Colour: green	Description: 'Four cheese margherita' and 'Mouth-watering pizza from Italy'<br>Image: Pizza with cheese (more than 2 and greasy) and very little tomato sauce<br>Colour: yellow
	Cake		
**No label (baseline and control)**	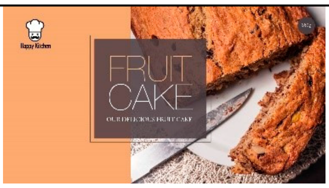	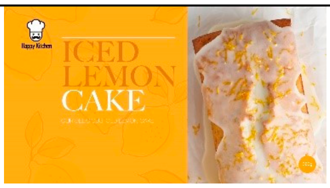	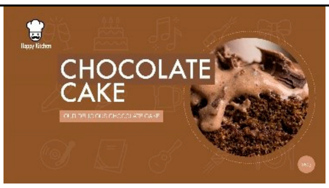

**Multiple Traffic Light**	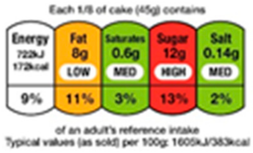	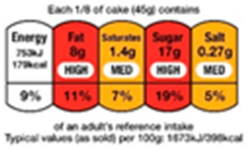	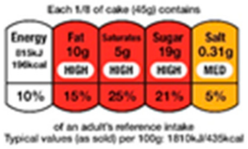
**Nutri-Score**	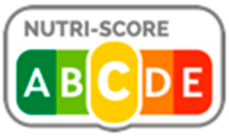	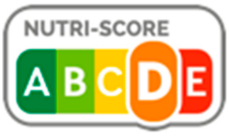	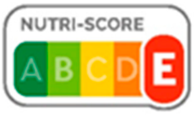
**Warning label**	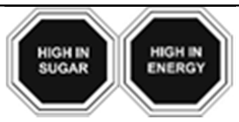	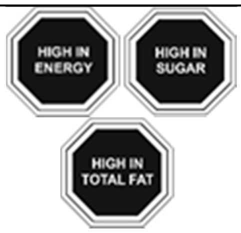	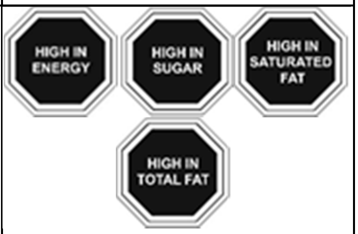
**Positive Choice tick**	N/A	N/A	N/A
**Design features**<br><br>All: size=360g (whole cake)	Description: 'Fruit cake' and 'Our delicious fruit cake'<br>Image: Plain loaf cake with fruit visible- no icing<br>Colour: Beige	Description: 'Iced lemon cake' and 'Our delicious lemon cake'<br>Image: Lemon cake loaf covered in icing and lemon zest<br>Colour: Yellow	Description: 'Chocolate cake' and 'Our delicious chocolate cake'<br>Image: Chocolate cake with chocolate icing and chunks<br>Colour: Brown
		**Crisps**	
**No label (baseline and control)**	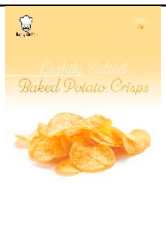	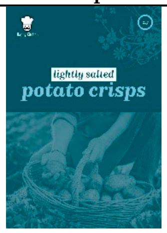	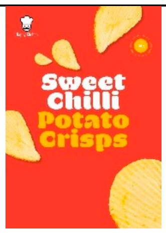
**Multiple Traffic Light**	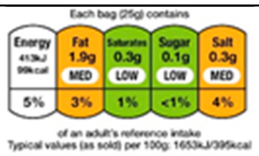	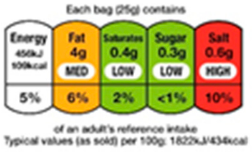	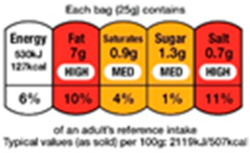
**Nutri-Score**	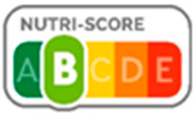	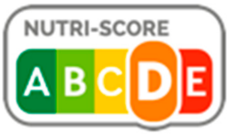	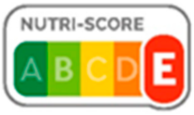
**Warning label**	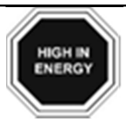	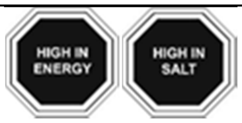	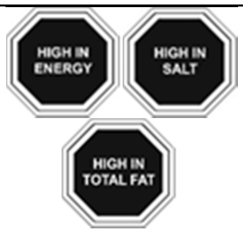
**Positive Choice tick**	N/A	N/A	N/A

**Design features**<br><br>All: size=25g (single packet)	Description: 'Lightly salted baked potato crisps' – italicised font<br>Image: pile of crisps<br>Colour: light yellow and white	Description: 'lightly salted potato crisps'- standard font<br>Image: Potatoes in a basket<br>Colour: blue	Description: 'Sweet chilli potato crisps'- bold font<br>Image: Large crisps<br>Colour: Bright red
		Yoghurt	
**No label (baseline and control)**	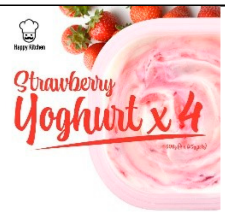	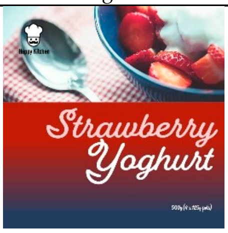	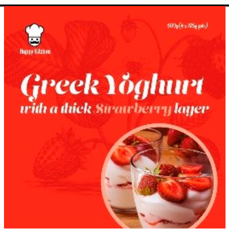
**Multiple Traffic Light**	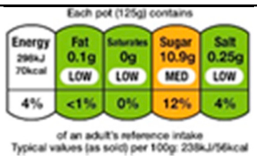	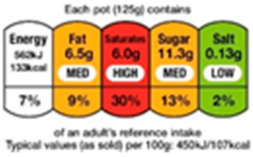	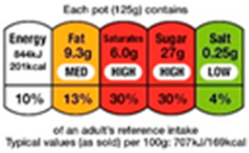
**Nutri-Score**	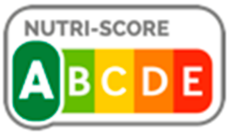	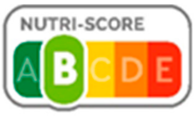	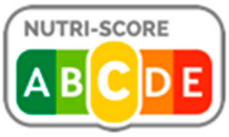
**Warning label**		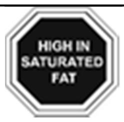	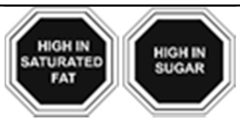
**Positive Choice tick**	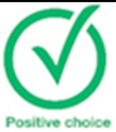		
**Design features**<br><br>All: size=500g (4x125g pots)	Description: 'Strawberry yoghurt'<br>Image: yoghurt with some strawberry mixed in a yoghurt pot and strawberries in the background<br>Colour: white	Description: 'Strawberry yoghurt'<br>Image: yoghurt with strawberries in a bowl<br>Colour: dull red	Description: 'Greek yoghurt with a thick strawberry layer'<br>Image: yoghurt with strawberries on top and strawberry compote layer in glasses<br>Colour: bright red
		**Breakfast cereal**	
**No label (baseline and control)**	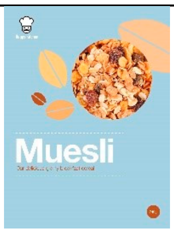	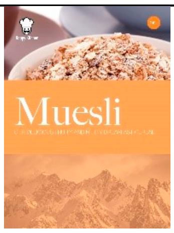	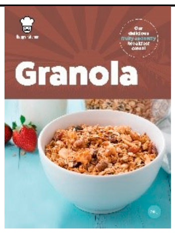
**Multiple Traffic Light**	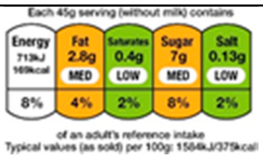	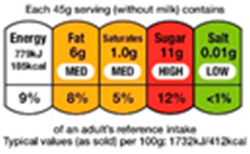	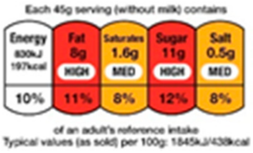

**Nutri-Score**	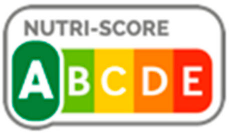	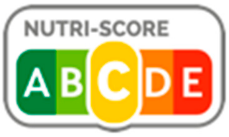	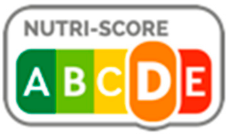
**Warning label**		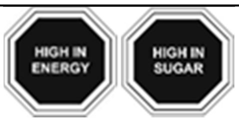	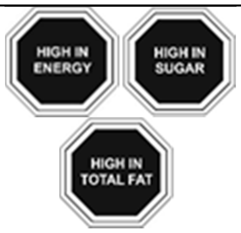
**Positive Choice tick**	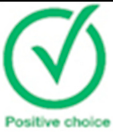		
**Design features**<br><br>All: size=740g	<u>Description</u>: 'Muesli' and 'Our delicious grainy breakfast cereal'<br><u>Image</u>: Muesli with plenty of wholegrain flakes and animated oats/nuts<br><u>Colour</u>: light blue	<u>Description</u>: 'Muesli' and 'Our delicious fruity and nutty breakfast cereal'<br><u>Image</u>: muesli in a bowl with more fruit and what could be sugar frosting and mountains to indicate swiss style muesli<br><u>Colour</u>: dull orange	<u>Description</u>: 'Granola' and 'Our delicious fruity and nutty breakfast cereal'<br><u>Image</u>: granola in a bowl<br><u>Colour</u>: dark brown
	**Instant hot chocolate powder**		
**No label (baseline and control)**	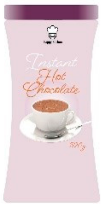	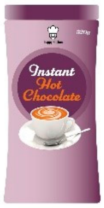	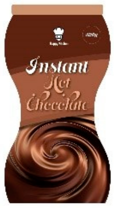
**Multiple Traffic Light**	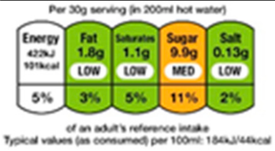	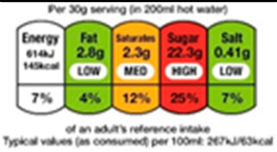	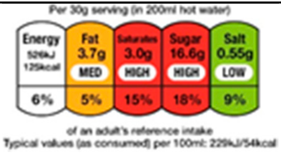
**Nutri-Score**	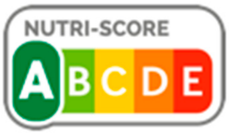	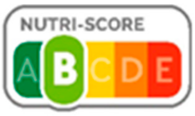	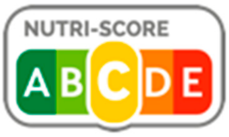
**Warning label**		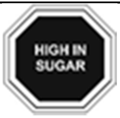	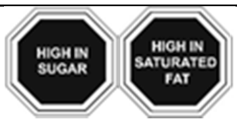
**Positive Choice tick**	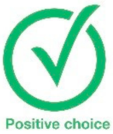		
**Design features**<br><br>All: size = 320g	<u>Description</u>: 'Instant Hot Chocolate' – thin font<br><u>Image</u>: plain hot chocolate in a mug	<u>Description</u>: 'Instant Hot Chocolate' – thicker font	<u>Description</u>: 'Instant Hot Chocolate'- cursive font<br><u>Image</u>: dark chocolate swirls

	Colour: light purple	Image: hot chocolate in a mug with a swirl of chocolate<br>Colour: darker purple	Colour: dark brown

Table S2. Nutritional specification of products (including information necessary for Multiple Traffic Light labels)

		Per package						Per 100g	
	**Portion size**	**kcal**	**kJ<br>%RI**	**Fat<br>%RI**	**Sat fat<br>%RI**	**Sugar<br>%RI**	**Salt<br>%RI**	**kJ**	**kcal**
**Pizza 1**	165	368	1549<br>18	9.9<br>14	3.3<br>17	6.4<br>7	1.16<br>19	933	223
**2**		404	1701<br>20	14<br>20	7.6<br>38	5.0<br>6	1.29<br>21	1031	245
**3**		469	1964<br>23	22<br>31	10.1<br>50	5.1<br>6	2.8<br>47	1188	284
**Cake 1**	45	172	722<br>9	8<br>11	0.6<br>3	12<br>13	0.14<br>2	1605	383
**2**		179	753<br>9	8<br>11	1.4<br>7	17<br>19	0.27<br>5	1673	398
**3**		196	815<br>10	10<br>15	5<br>25	19<br>21	0.31<br>5	1810	435
**Crisps 1**	25	99	413<br>5	1.9<br>3	0.3<br>1	0.1<br>0	0.3<br>4	1653	395
**2**		109	456<br>5	4<br>6	0.4<br>2	0.3<br><1%	0.6<br>10	1822	434
**3**		127	530<br>6	7<br>10	0.9<br>4	1.3<br>1	0.7<br>11	2119	507
**Yoghurt 1**	125	70	298<br>4	0.1<br><1%	0<br>0	10.9<br>12	0.25<br>4	238	56
**2**		133	562<br>7	6.5<br>9	6.0<br>30	11.3<br>13	0.13<br>2	450	107
**3**		201	844<br>10	9.3<br>13	6.0<br>30	27<br>30	0.25<br>4	707	169
**Breakfast cereal 1**	45	169	713<br>8	2.8<br>4	0.4<br>2	7<br>8	0.13<br>2	1584	375
**2**		185	779<br>9	6<br>8	1.0<br>5	11<br>12	0.01<br>0	1732	412
**3**		197	830<br>10	8<br>11	1.6<br>8	11<br>12	0.5<br>8	1845	438
**Hot Choc 1**	230ml (30g powder + 200 ml water)	101	422<br>5	1.8<br>3	1.1<br>5	9.9<br>11	0.13<br>2	184	44
**2**		145	614<br>7	2.8<br>4	2.3<br>12	22.3<br>25	0.41<br>7	267	63
**3**		125	526<br>6	3.7<br>5	3.0<br>15	16.6<br>18	0.55<br>9	229	54

Kcal: Kilocalorie; kJ: Kilojoule; RI: Reference Intakes.

Table S3. Individual characteristics of the analysis sample, by experimental group

	**Control (*n* = 913)**	**MTL (*n* = 907)**	**N-S (*n* = 924)**	**WL (*n* =895)**	**PC (*n*= 891)**	**Overall (*n* =4,530)**
	***n* (%)**	***n* (%)**	***n* (%)**	***n* (%)**	***n* (%)**	***n* (%)**
**Sex**						
Female	518 (56.7)	512 (56.5)	534 (57.8)	510 (57.0)	512 (57.5)	2,586 (57.1)
Male	395 (43.3)	395 (43.6)	390 (42.2)	385 (43.0)	379 (42.5)	1,944 (42.9)

**Age**						
18-29	61 (6.7)	65 (7.2)	56 (6.1)	56 (6.3)	41 (4.6)	279 (6.2)
30-39	139 (15.2)	101 (11.2)	141 (15.3)	132 (14.8)	129 (14.5)	642 (14.2)
40-49	169 (18.5)	181 (20.0)	171 (18.5)	180 (20.1)	180 (20.2)	881 (19.5)
50-59	199 (21.8)	204 (22.5)	187 (20.2)	185 (20.7)	194 (21.8)	969 (21.4)
60-69	179 (19.6)	191 (21.1)	197 (21.3)	181 (20.3)	185 (20.8)	933 (20.6)
70+	166 (18.2)	164 (18.1)	172 (18.6)	160 (17.9)	161 (18.1)	823 (18.2)
**Ethnicity**						
White British	799 (87.5)	789 (87.0)	809 (87.6)	792 (88.5)	785 (88.1)	3,974 (87.7)
White other	53 (5.8)	55 (6.1)	51 (5.5)	50 (5.6)	50 (5.6)	259 (5.7)
Mixed or multiple ethnic groups	9 (1.0)	13 (1.4)	15 (1.6)	6 (0.7)	11 (1.2)	54 (1.2)
Asian or Asian British	37 (4.1)	33 (3.6)	28 (3.0)	32 (3.6)	25 (2.8)	155 (3.4)
Black or Black British	12 (1.3)	14 (1.5)	17 (1.8)	13 (1.5)	16 (1.8)	72 (1.6)
Other	3 (0.3)	3 (0.3)	4 (0.4)	2 (0.2)	4 (0.5)	16 (0.4)
**Education**						
Degree or equivalent +	460 (50.4)	435 (48.0)	450 (48.7)	426 (47.6)	425 (47.7)	2,196 (48.5)
A levels or vocational level 3 or equivalent	174 (19.1)	174 (19.2)	179 (19.4)	174 (19.4)	177 (19.9)	878 (19.4)
Other qualifications below A levels or equivalent	163 (17.9)	155 (17.1)	167 (18.1)	158 (17.7)	141 (15.8)	784 (17.3)
Other qualification	46 (5.0)	70 (7.7)	44 (4.8)	49 (5.5)	62 (7.0)	271 (6.0)
No qualifications	70 (7.7)	73 (8.1)	84 (9.1)	88 (9.8)	86 (9.7)	401 (8.9)
**Children in household**						
Yes	280 (30.7)	262 (28.9)	269 (29.1)	286 (32.0)	280 (31.4)	1,377 (30.4)
No	633 (69.3)	645 (71.1)	655 (70.9)	609 (68.0)	611 (68.6)	3,153 (69.6)
**Shopping responsibility**						
Yes – some or all	868 (95.0)	866 (95.5)	895 (96.9)	859 (96.0)	852 (95.6)	4,340 (95.8)
No – someone else does	45 (4.9)	41 (4.5)	29 (3.1)	36 (4.0)	39 (4.4)	190 (4.2)
**Current label use**						
Very often	177 (19.4)	209 (23.0)	179 (19.4)	220 (24.6)	160 (18.0)	945 (20.9)
Quite often	297 (32.5)	345 (38.0)	305 (33.0)	308 (34.4)	291 (32.7)	1,546 (34.1)
Occasionally	269 (29.5)	234 (25.8)	267 (28.9)	266 (29.7)	282 (31.7)	1,318 (29.1)
Rarely	132 (14.5)	95 (10.5)	142 (15.4)	83 (9.3)	107 (12.0)	559 (12.3)
Never	38 (4.2)	24 (2.7)	31 (3.4)	18 (2.0)	51 (5.7)	162 (3.6)
**Reported consuming or buying product in past 12 months**						
Pizza	670 (73.4)	647 (71.3)	686 (74.2)	690 (77.1)	668 (75.0)	3,361 (74.2)
Drink	313 (34.3)	336 (37.1)	324 (35.1)	336 (37.5)	321 (36.0)	1,630 (36.0)
Cake	627 (68.7)	651 (71.8)	633 (68.5)	635 (71.0)	617 (69.3)	3,163 (69.8)
Crisps	752 (82.4)	739 (81.5)	772 (83.6)	753 (84.1)	707 (79.4)	3,723 (82.2)
Yoghurt	761 (83.4)	755 (83.2)	772 (83.6)	747 (83.5)	744 (83.5)	3,779 (83.4)
Breakfast cereal	779 (85.3)	757 (83.5)	755 (81.7)	756 (84.5)	755 (84.7)	3,802 (83.9)

MTL, Multiple Traffic Light; N-S, Nutri-Score; WL, Warning Label; PC, Positive Choice tick; RR: Relative Risk; CI: Confidence Interval.

Table S4. Proportion of missing data per variable (*n* = 4,863)

	**n (%)**
Sex	0 (0)
Age	6 (0.1)
Ethnicity	58 (1.6)
Education	16 (0.3)
Children in household	21 (0.4)
Shopping responsibility	1 (0.0)
Current label use	0 (0.0)
Reported consuming or buying product in past 12 months*	0 (0.0)

Baseline ranking	
Pizza	55 (1.1)
Drink	98 (2.0)
Cake	53 (1.1)
Crisps	47 (1.0)
Yoghurt	71 (1.5)
Breakfast cereal	56 (1.2)
**Follow-up ranking**	
Pizza	48 (1.0)
Drink	55 (1.1)
Cake	40 (0.8)
Crisps	38 (0.8)
Yoghurt	49 (1.0)
Breakfast cereal	51 (1.0)
**Enough information question**	
Baseline	20 (0.4)
Follow-up	27 (0.6)
Reported seeing the label	2 (0.0)

Missing data includes the following responses: refused, don't know, not applicable

Table S5. Summary of participants who correctly ranked products at baseline and follow-up, by FOPL group and product category

	Control % correct		MTL % correct		NS % correct		WL % correct		PC % correct		Overall % correct	
	**Baseline**	**Follow-up**	**Baseline**	**Follow-up**	**Baseline**	**Follow-up**	**Baseline**	**Follow-up**	**Baseline**	**Follow-up**	**Baseline**	**Follow-up**
**Pizza**	465 (69.4%)	456 68.1%)	473 (73.1%)	582 (90.0 %)	508 (74.1%)	640 (93.3 %)	476 (69.0%)	563 (81.6 %)	490 (73.4%)	491 (73.5 %)	2412 (71.8%	2732 (81.3 %)
**Drinks**	198 (63.3%)	196 (62.6)	212 (63.1%)	286 (85.1 %)	183 (56.5%)	306 (94.4 %)	221 (65.8%)	280 (83.3 %)	194 (60.4)%	221 (68.9 %)	1008 (61.8%)	1289 (79.1 %)
**Puddings**	422 (67.3%)	417 (66.5 %)	428 (65.8%)	593 (91.1 %)	445 (70.3%)	590 (93.2 %)	399 (62.8%)	547 (86.1 %)	422 (68.4%)	411 (66.6 %)	2116 (66.9%)	2558 (80.9 %)
**Snack**	453 (60.2%)	459 (61.0 %)	468 (63.3%)	675 (91.3 %)	478 (61.9%)	723 (93.7 %)	504 (66.9%)	629 (83.5 %)	454 (64.2%)	464 (65.6 %)	2357 (63.3%)	2950 (79.2 %)
**Yoghurt**	98 (12.9 %)	101 (13.3 %)	107 (14.2%)	642 (85.0 %)	86 (11.1%)	660 (85.5 %)	90 (12.1%)	458 (61.3 %)	66 (8.9%)	117 (15.7 %)	447 (11.8%)	1978 (52.3 %
**Breakfast cereal**	261 (33.5%)	267 (34.3 %)	252 (33.3%)	658 (86.9 %)	256 (33.9%)	664 (88.0 %)	238 (31.5%)	546 (72.2 %)	261 (34.6%)	299 (39.6 %)	1268 (33.4%)	2434 (64.0 %)

FOPL: front of pack label; MTL, Multiple Traffic Light; N-S, Nutri-Score; WL, Warning Label; PC, Positive Choice tick.

Table S6. Multilevel log-binomial regression results - follow-up (FOPL group) correct (yes /no) and adjusted for baseline rank compared to control (also adjusted for design factors and covariates as planned) in full sample (*n* = 4,530)

	MTL v Control RR (95%CI)	N-S v Control RR (95%CI)	WL v Control RR (95%CI)	PT v Control RR (95%CI)	N-S v MTL RR (95%CI)

**Pizza**	1.19<br>(1.09, 1.31)<br>***p* < 0.001**	1.26<br>(1.16, 1.38)<br>***p* < 0.001**	1.15<br>(1.05, 1.25)<br>***p* < 0.01**	0.98<br>(0.91, 1.06)<br>0.63	1.06<br>(0.97, 1.16)<br>*p* = 0.22
**Drink**	1.37<br>(1.24, 1.51)<br>***p* < 0.001**	1.66<br>(1.51, 1.83)<br>***p* < 0.001**	1.29<br>(1.18, 1.40)<br>***p* < 0.001**	1.13<br>(1.04, 1.23)<br>***p* < 0.01**	1.21<br>(1.09, 1.35)<br>***p* < 0.01**
**Cake**	1.41<br>(1.30, 1.54)<br>***p* < 0.001**	1.44<br>(1.33, 1.57)<br>***p* < 0.001**	1.37<br>(1.26, 1.48)<br>***p* < 0.001**	1.01†<br>(0.95, 1.08)<br>0.76	1.02<br>(0.93, 1.12)<br>*p* = 0.70
**Crisps**	1.43<br>(1.31, 1.56)<br>***p* < 0.001**	1.50<br>(1.37, 1.64)<br>***p* < 0.001**	1.27<br>(1.17, 1.38)<br>***p* < 0.001**	1.02†<br>(0.95, 1.10)<br>0.59	1.05<br>(0.95, 1.16)<br>*p* = 0.38
**Yoghurt**	6.19<br>(4.79, 8.01)<br>***p* < 0.001**	6.86<br>(5.14, 9.24)<br>***p* < 0.001**	4.24<br>(3.15, 5.71)<br>***p* < 0.001**	1.79<br>(1.35, 2.37)<br>***p* <0.001**	1.11<br>(0.80, 1.55)<br>*p* = 0.53
**Breakfast cereal**	2.51<br>(2.14, 2.95)<br>***p* < 0.001**	2.61<br>(2.21, 3.08)<br>***p* < 0.001**	2.43<br>(2.02, 2.93)<br>***p* < 0.001**	1.17<br>(0.99, 1.37)<br>P = 0.06	1.04<br>(0.87, 1.24)<br>*p* = 0.69

†Cake and crisps categories had no products qualify for Positive Choice tick; all analyses were adjusted for baseline ranking (correct/ incorrect), stratification factors (year of recruitment to panel, sex, age, government office region, household income), and the following pre-specified covariates: ethnicity, highest education level, household composition, food shopping responsibility, and current FOPL use. FOPL: front of pack label; MTL, Multiple Traffic Light; N-S, Nutri-Score; WL, Warning Label; PC, Positive Choice tick; RR: Relative Risk; CI: Confidence Interval.

Table S7. Log-binomial regression results – relative risk that ranking improved (follow-up vs baseline) between FOPL group and control (adjusted for design factors and covariates as planned) in full sample (*n* = 4,530)

	MTL v Control<br>RR (95%CI)	N-S v Control<br>RR (95%CI)	WL v Control<br>RR (95%CI)	PC v Control<br>RR (95%CI)	N-S v MTL<br>RR (95%CI)
**Pizza**	2.23<br>(1.56, 3.17)<br>***p* < 0.001**	2.46<br>(1.74, 3.48)<br>***p* < 0.001**	1.95<br>(1.36, 2.80)<br>***p* < 0.001**	0.86<br>(0.58, 1.28)<br>0.46	1.11<br>(0.88, 1.39)<br>P = 0.40
**Drink**	4.04<br>(2.73, 5.96)<br>***p* < 0.001**	5.32<br>(3.64, 7.76)<br>***p* < 0.001**	3.29<br>(2.22, 4.90)<br>***p* < 0.001**	1.82<br>(1.18, 2.81)<br>***p* < 0.01**	1.32<br>(1.10, 1.58)<br>**v < 0.01**
**Cake**	8.05<br>(4.83, 13.44)<br>***p* < 0.001**	8.02<br>(4.84, 13.30)<br>***p* < 0.001**	7.06<br>(4.24, 11.76)<br>***p* < 0.001**	1.00†<br>(0.51, 1.97)<br>0.99	0.99<br>(0.81, 1.22)<br>*p* = 0.97
**Crisps**	4.75<br>(3.29, 6.84)<br>***p* < 0.001**	5.35<br>(3.72, 7.69)<br>***p* < 0.001**	3.59<br>(2.45, 5.26)<br>***p* < 0.001**	1.36†<br>(0.85, 2.20)<br>0.20	1.13<br>(0.94, 1.35)<br>*p* = 0.20
**Yoghurt**	22.70<br>(14.72, 34.99)<br>***p* < 0.001**	22.89<br>(14.84, 35.30)<br>***p* < 0.001**	15.63<br>(10.08, 24.24)<br>***p* < 0.001**	2.84<br>(1.70, 4.73)<br>***p* < 0.001**	1.01<br>(0.93, 1.10)<br>*p* = 0.85
**Breakfast cereal**	7.64<br>(5.44, 10.73)<br>***p* < 0.001**	8.37<br>(5.98, 11.73)<br>***p* < 0.001**	6.90<br>(4.90, 9.73)<br>***p* < 0.001**	2.05<br>(1.37, 3.05)<br>***p* < 0.001**	1.10<br>(0.98, 1.23)<br>*p* = 0.13

†Cake and crisps categories had no products qualify for Positive Choice tick; all analyses were adjusted for the five stratification factors (year of recruitment to panel, sex, age, government office region, household income) and the following pre-specified covariates: ethnicity, highest education level,

household composition, food shopping. FOPL: front of pack label; MTL, Multiple Traffic Light; N-S, Nutri-Score; WL, Warning Label; PC, Positive Choice tick; RR: Relative Risk; CI: Confidence Interval.

Table S8. Multiple regression analysis results- association between global food score and FOPL group (adjusted for design factors and covariates as planned) in full sample (*n* = 4,530)

	MTL v Control RR (95%CI)	N-S v Control RR (95%CI)	WL v Control RR (95%CI)	PC v Control RR (95%CI)	N-S v MTL RR (95%CI)
**Score (-5, +5)**	1.8	2.0	1.4	0.1	0.3
**Regression**	(1.7, 1.9)	(1.9, 2.1)	(1.2, 1.5)	(0.02, 0.2)	(0.2, 0.5)
**(coefficients)**	$p < 0.001$	$p < 0.001$	$p < 0.001$	$p = 0.03$	$p < 0.001$

Global food score was an aggregated score of correct ranking in the five food products, range -5 to +5 - indicates worsening and + indicates improvement) ; all analyses adjusted for stratification factors (year of recruitment to panel, sex, age, government office region, household income) and covariates: ethnicity, highest education level, household composition, food shopping responsibility, current FOPL use. FOPL: front of pack label; MTL, Multiple Traffic Light; N-S, Nutri-Score; WL, Warning Label; PC, Positive Choice tick; RR: Relative Risk; CI: Confidence Interval.
